# The International Collaborative Gaucher Group GRAF (Gaucher Risk Assessment for Fracture) score: a composite risk score for assessing adult fracture risk in imiglucerase-treated Gaucher disease type 1 patients

**DOI:** 10.1186/s13023-020-01656-6

**Published:** 2021-02-18

**Authors:** Patrick Deegan, Aneal Khan, José Simon Camelo, Julie L. Batista, Neal Weinreb

**Affiliations:** 1 Lysosomal Disorders Unit, Addenbrooke’s Hospital, Cambridge University Hospitals NHS Foundation Trust, Hills Road, Box 135, Cambridge, CB2 0QQ UK; 2 Department of Medical Genetics and Pediatrics, Alberta Children’s Hospital Research Institute, Cumming School of Medicine, University of Calgary, Calgary, Canada; 3 Department of Pediatrics, Ribeirão Preto Medical School, University of São Paulo, São Paulo, Brazil; 4 Sanofi Genzyme, Cambridge, MA USA; 5 Departments of Human Genetics and Medicine (Hematology), University of Miami Miller School of Medicine, Miami, FL USA

**Keywords:** Gaucher disease, Fracture, Imiglucerase, Enzyme replacement therapy, Risk factor

## Abstract

**Background:**

Fractures in Gaucher disease type 1 (GD1) patients cause significant morbidity. Fracture risk may be decreased by enzyme replacement therapy (ERT) but not eliminated. When considering initiation of treatment, it is useful to know to what extent fixed patient-specific factors determine risk for future fractures beyond standard risk factors that change with time and treatment, such as decreased bone mineral density. We developed a tool called the GRAF score (Gaucher Risk Assessment for Fracture) that applies 5 widely available characteristics (sex, age at treatment initiation [ATI], time interval between diagnosis and treatment initiation, splenectomy status, history of pre-treatment bone crisis) and provides a practical method to assess future fracture risk when imiglucerase ERT is initiated.

**Methods:**

Inclusion criteria: GD1 patients in the International Collaborative Gaucher Group Gaucher Registry as of September 2019 initially treated with alglucerase/imiglucerase; known splenectomy status; at least one skeletal assessment on treatment (3216 of 6422 patients). Data were analyzed by ATI group (< 18, ≥ 18 to < 50, or ≥ 50 years of age) using Cox proportional hazards regression with all 5 risk factors included in the multivariable model. A composite risk score was calculated by summing the contribution of each parameter weighted by the strength of its association (regression coefficient) with fracture risk.

**Results:**

Patients were followed from the date of treatment initiation (or age 18 years for patients if treatment started earlier) to the date of first adult fracture (n = 288 first fracture endpoints), death, or end of follow-up. The GRAF score for each ATI group was associated with a 2.7-fold increased risk of adult fracture for each one-point increase (*p* < 0.02 for < 18 ATI, *p* < 0.0001 for ≥ 18 to < 50 ATI and ≥ 50 ATI).

**Conclusions:**

The GRAF score is a tool to be used with bone density and other modifiable, non-GD-specific risk factors (e.g. smoking, alcohol intake, frailty) to inform physicians and previously untreated GD1 patients about risk for a future fracture after starting imiglucerase regardless of whether there is an eventual switch to an alternative ERT or to substrate reduction therapy. GRAF can also help predict the extent that fracture risk increases if initiation of treatment is further delayed.

## Introduction

Gaucher disease (GD) is a lysosomal storage disease caused by mutations in the glucocerebrosidase gene (*GBA1*) that leads to deficient lysosomal acid β-glucosidase (glucocerebrosidase, EC 3.2.1.21) activity. GD has classically been categorized into 3 main types (GD types 1, 2 and 3). The majority of cases are classified as GD type 1 (GD1), which is characterized by a lack of early-onset central nervous system disease, unlike GD types 2 and 3. Patients with GD1 can present with hepatosplenomegaly, anemia, thrombocytopenia, bone pain, growth failure in childhood and a number of additional manifestations [1, 2]. Skeletal manifestations of GD1 are significant sources of pain and disability, including bone pain, bone crisis, osteonecrosis, lytic lesions, and fracture [3–7]. GD1 affects both supportive bone tissue and bone marrow.

GD-related changes at the cellular level contribute to decreased bone accretion in children, decreased peak bone mass in young adults, and the further development of low bone mass with age. Low bone mass is the strongest factor affecting fracture risk in the general population and is also associated with fracture in GD [6, 8]. Some characteristics distinguish GD1 fractures from the general population. The most common site of fracture in GD1 is the spine followed by the hip and appendicular skeleton whereas the hip is the most common site of post-menopausal osteoporosis [7]. Other factors specific to GD1, such as cortical bone thinning, osteolytic lesions and focal disruption of bone architecture, may be associated with fracture [3, 6, 7, 9]. A direct evaluation of clinical risk factors for fracture in GD, other than bone mineral density (BMD), is thus warranted.

Fractures are known to cause morbidity and reduced quality of life in GD1 patients [3, 10]. In a previous study of 1698 GD1 patients enrolled in the International Collaborative Gaucher Group (ICGG) Gaucher Registry (NCT00358943), 15% had fractures [1]. In a multi-center study in the United Kingdom (UK), 28% reported fragility fractures [10]. In a cohort of 105 adult GD1 patients in France, 18% had non-vertebral fractures and there was a 15% prevalence of vertebral fractures [11]. Non-vertebral fractures were significantly greater in splenectomized patients, similar to results from the UK study. The mean age at vertebral fracture was 47 years with a range of 29 to 70 years.

Studies on fractures in patients with GD have improved our understanding of the risk factors for fracture. As with the general population [12], a significant determinant of fracture risk in GD is bone density as measured by dual energy X-ray absorptiometry (DXA), with a 5.55 times higher risk of fracture at any site when the spine Z-score is less than or equal to  −1 [5]. This study exposed a lower-threshold DXA bone density for fracture compared to the general population suggesting that additional GD-specific factors exist. Bone disease in GD patients can be occult and aggravated by previous splenectomy [13] and delays in diagnosis and treatment initiation [14]. Thus, significant bone disease may be present by the time disease-modifying therapy is started [10, 11, 14–16].

Although DXA scanning is recommended in several GD management guidelines [17], not all GD1 patients worldwide have ready access to DXA. The impact of common demographic features readily available at clinic visits (e.g. sex, year of birth, treatment status, and splenectomy status) have only been assessed in smaller studies [5]. The aim of this study, therefore, was to create a composite risk score assessment (the Gaucher Risk Assessment for Fracture [GRAF]) as a clinical tool to be used with other modifiable, non-GD-specific risk factors (e.g. smoking, alcohol intake, frailty) to inform physicians and previously untreated adult GD1 patients about risk for a future fracture after starting imiglucerase. While there are risk fracture scores (e.g., FRAX® [18]) available for the general population, a tool specific to GD-related factors would be additionally beneficial for the management of GD1 patients.

## Results

### Patient disposition for study population

As of 06 September 2019, 3216 (50.1%) of 6422 patients in the ICGG Gaucher Registry met all inclusion and no exclusion criteria for the study. Excluded patients: 865 (13.5%) patients were not specifically reported in the Registry as diagnosed as GD1; 191 (3.0%) patients had no diagnosis date; 1031 (16.1%) were excluded because treatment status was unknown or they were reported as never treated; 502 (7.8%) patients’ initial treatment was not imiglucerase; 16 (0.2%) patients had no recorded splenectomy status and 33 (0.5%) splenectomized patients did not have a splenectomy date; 524 (8.2%) patients did not have a complete medical history or at least one skeletal assessment; 44 (0.7%) patients with a reported fracture did not have a fracture date.

Of the 3216 study population patients, 568 were excluded from the fracture risk analysis population because they reported a first fracture prior to age 18 (n = 104), reported a first fracture prior to initiation of treatment (n = 125), or had no follow-up after age 18 (n = 339). Thus, the fracture risk analysis population was 2648 patients of whom 288 had a first-time fracture in adulthood after imiglucerase was started.

### Demographic characteristics and distribution of first fracture location among patients with fracture in the total study population

Summary data on the demographic and clinical characteristics of patients with fracture (pediatric and adult) and the location of first fractures (pediatric and adult) are shown in supplemental tables, stratified by whether first fracture occurred before or after initiation of imiglucerase (Additional file 2: Table S-II, Additional file 3: Table S-III, Additional file 4: Table S-IV, Additional file 5: Table S-V).

### Baseline demographic and clinical characteristics among patients with and without fracture in the fracture risk analysis population

The total person-years of follow-up (i.e., total follow-up time across all patients at risk of fracture) for the analysis population was 30,666 person-years, with individual patients being followed for a median (25%, 75%) of 10.9 (4.6, 17.8) years. During this time, 288 first fractures in adulthood were reported, resulting in an overall incidence rate of 9.4 fractures per 1000 person-years. Fifty of the fractures occurred after switching from imiglucerase to another therapy and 84 fractures occurred on or after 25 June 2009 (the beginning of a temporary imiglucerase shortage). The mean (standard deviation [SD]) age of first fracture during adulthood for those 288 patients with fracture was 49.2 (16.6) years and ranged from 18.1 to 90.4 years (Additional file 3: Table S-III). Additionally, the frequencies of multiple fractures reported on the first fracture date and of subsequent fractures reported after the first fracture date are shown in supplemental materials (Additional file 6: Table S-VI).

Sex, genotype (using legacy nomenclature: N370S homozygous, all other genotypes, unknown genotype) and other baseline demographic and clinical characteristics for the fracture risk analysis population (with and without fracture during follow-up) by ATI cohort are summarized in Table 1. The majority of patients in the analysis population were in the ≥ 18 to < 50 year ATI cohort and did not report a fracture in adulthood. Within each ATI cohort, patients with fracture tended to be older at treatment initiation, have a longer time interval between diagnosis and treatment initiation, and were more likely to have a history of splenectomy and bone crisis prior to treatment initiation than those without fracture. In all ATI cohorts, women, pre- and post-menopausal, had more fractures than men.

**Table 1:** Demographic and clinical characteristics in the fracture risk analysis population by ATI and fracture status

Parameter	< 18 years ATI cohort		18 to < 50 years ATI cohort		>= 50 years ATI cohort	
	Patients with fracture	Patients without fracture	Patients with fracture	Patients without fracture	Patients with fracture	Patients without fracture
Number of patients, n						
	33	623	167	1318	88	419
Sex, n (%)						
Male	14 (42.4)	297 (47.7)	72 (43.1)	569 (43.2)	30 (34.1)	241 (57.5)
Female	19 (57.6)	326 (52.3)	95 (56.9)	749 (56.8)	58 (65.9)	178 (42.5)
Age at GD1 diagnosis, years						
Mean (SD)	8.3 (4.64)	7.0 (4.33)	20.5 (13.44)	23.2 (12.65)	45.7 (18.26)	38.8 (20.45)
Median (25th, 75th)	6.9 (4.6, 11.8)	6.1 (3.6, 10.0)	20.8 (7.5, 29.3)	23.5 (12.8, 32.6)	50.1 (32.9, 58.3)	39.8 (23.2, 55.3)
Range	2.9, 17.6	0.0, 17.4	0.0, 49.3	0.0, 49.7	0.5, 81.8	2.3, 78.9
Age at imiglucerase initiation, years						
Mean (SD)	12.0 (4.61)	10.0 (4.48)	36.6 (8.22)	33.8 (9.03)	60.5 (8.00)	60.9 (8.32)
Median (25th, 75th)	14.1 (8.3, 15.2)	10.2 (6.1, 13.8)	37.5 (30.3, 43.7)	33.8 (26.5, 41.4)	59.0 (53.8, 65.6)	59.3 (54.4, 65.7)
Range	3.1, 17.9	0.2, 18.0	18.3, 49.9	18.0, 50.0	50.0, 84.1	50.2, 84.2
Time interval between diagnosis and imiglucerase initiation, years						
Mean (SD)	3.7 (4.36)	2.9 (3.79)	16.1 (12.09)	10.6 (11.02)	14.9 (15.95)	22.1 (18.59)
Median (25th, 75th)	0.9 (0.3, 7.7)	1.0 (0.3, 4.6)	17.5 (2.9, 25.0)	6.8 (0.7, 18.0)	8.9 (0.8, 26.7)	18.4 (1.4, 39.1)
Range	0.1, 12.9	0.0, 16.6	0.0, 44.0	0.0, 46.5	0.0, 61.4	0.0, 60.6
Genotype, n (%)						
N370S/N370S	6 (18.2)	65 (10.4)	35 (21.0)	309 (23.4)	43 (48.9)	166 (39.6)
All other genotypes	24 (72.7)	481 (77.2)	116 (69.4)	775 (58.8)	34 (38.6)	179 (42.7)
Unknown genotype	3 (9.1)	77 (12.4)	16 (9.6)	234 (17.8)	11 (12.5)	74 (17.7)
Splenectomized prior to or on treatment initiation date, n (%)						
No	27 (81.8)	567 (91.0)	88 (52.7)	942 (71.5)	56 (63.6)	299 (71.4)
Yes	6 (18.2)	56 (9.0)	79 (47.3)	376 (28.5)	32 (36.4)	120 (28.6)
Age at splenectomy, years						
n	6	56	79	376	32	120
Mean (SD)	8.7 (4.84)	7.7 (3.97)	17.5 (11.27)	19.4 (10.74)	27.2 (13.78)	36.6 (16.62)
Median (25th, 75th)	8.3 (3.8, 12.6)	7.5 (4.3, 10.1)	14.1 (8.0, 25.3)	18.5 (10.4, 26.5)	27.2 (14.2, 37.1)	37.8 (23.0, 50.1)
Range	3.7, 15.6	2.1, 17.3	1.7, 45.0	1.5, 48.8	5.0, 57.1	4.5, 72.9
Bone crisis reported prior to or on treatment initiation date, n (%)						
No	30 (90.9)	555 (89.1)	134 (80.2)	1201 (91.1)	77 (87.5)	398 (95.0)
Yes	3 (9.1)	68 (10.9)	33 (19.8)	117 (8.9)	11 (12.5)	21 (5.0)

ATI = Age at treatment initiation; GD1 = Gaucher disease type 1

^*^Excludes patients from total study sample with first fracture prior to treatment initiation, first fracture prior to age 18, and patients whose follow-up did not extend past age 18 years

### Fracture risk analyses

**Fig. 1:**
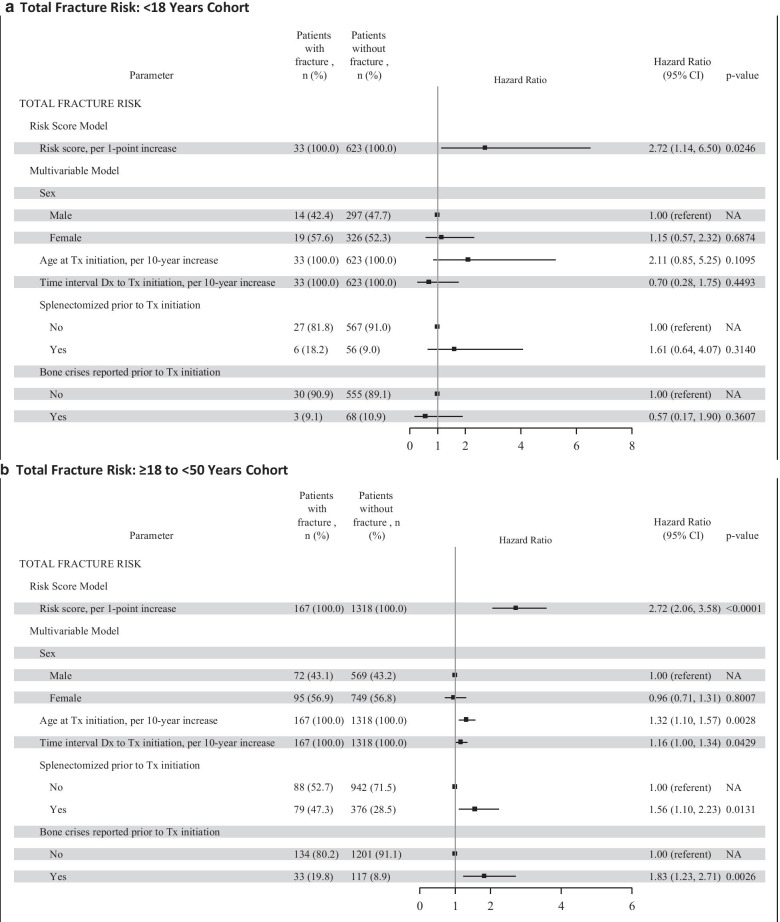

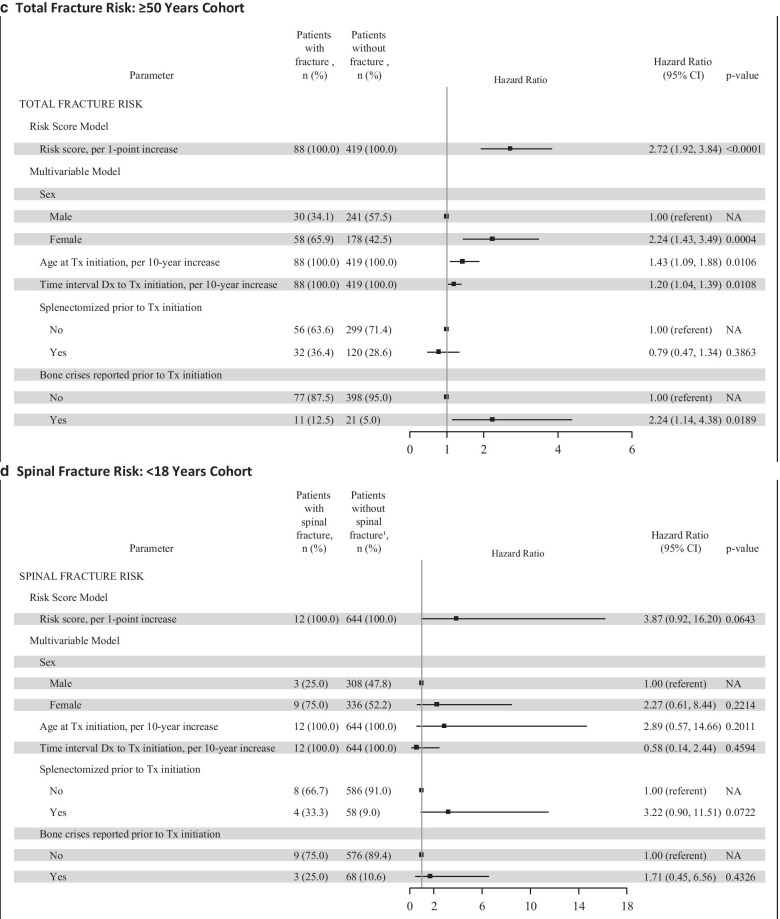

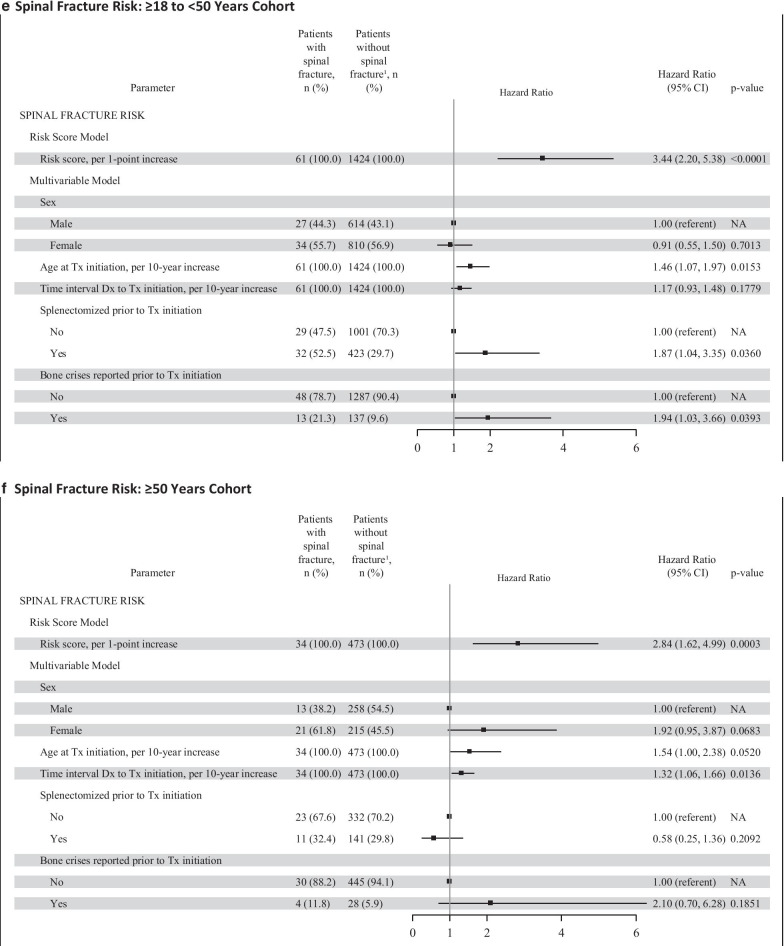
Total GRAF Score by ATI Cohort. The total risk score (Gaucher Risk Assessment for Fracture [GRAF] score) and the individual risk factor estimates from the Cox proportional hazards regression for first fracture (any type) in adulthood after treatment initiation are plotted by each age at treatment initiation (ATI) cohort in panels A through C. Panel A includes the forest plots for the < 18 years ATI cohort. Panel B includes the forest plots for the ≥ 18 to < 50 years ATI cohort. Panel C includes the forest plots for the ≥ 50 years ATI cohort. Panels D through F are the corresponding forest plots of the GRAF score and individual risk factor estimates for the secondary spinal fractures analyses. CI = confidence interval; Dx = diagnosis; NA = not applicable; Tx = treatment

The results of the Cox proportional hazard regression by ATI cohort for all types of fractures and spinal fractures separately are presented in Fig. 1 as Forest plots. The multivariable model for total fracture risk showed variation in statistical significance and magnitude of the hazard ratios for the 5 individual risk factors across the 3 ATI cohorts. For the < 18 year ATI cohort, all types of fracture (panel A), no individual factor was statistically significant, although the overall risk score was statistically significant (*p* < 0.05). For the ≥ 18 to < 50 year ATI cohort, all types of fracture (panel B), all factors except sex were statistically significant (*p* < 0.05). Older age at treatment initiation, longer interval between diagnosis and treatment initiation, having been splenectomized, and having bone crisis prior to treatment initiation were associated with greater fracture risk. For the ≥ 50 year ATI cohort, all types of fracture (panel C), sex, age at treatment initiation, time interval between diagnosis and treatment, and having had a bone crisis were statistically significant factors (*p* < 0.01); splenectomy status was not statistically significant. The individual risk factor estimates within each ATI cohort for spinal fracture were similar in magnitude and direction as for total fracture risk; however, several factors did not reach statistical significance, likely due to fewer spinal fracture endpoints (Fig. 1, panels D to F).

Summary statistics of the length of follow-up and the GRAF score values as well as the relationship between GRAF score and fracture risk are presented by ATI cohort in Table 2. Median follow-up time was 7.9, 13.8, and 8.2 years in the < 18, ≥ 18 to < 50, and ≥ 50 year ATI cohorts, respectively. The median GRAF score values in the risk analysis population from the younger ATI cohort to the older ATI cohort were 0.7, 1.2, and 2.8, respectively. The median GRAF score values for the subset of patients with fracture in each ATI cohort (youngest to oldest) were 0.8, 1.5, and 3.1, respectively.

**Table 2:** Duration of follow-up, GRAF score, and association of GRAF score with fracture risk, by ATI

	N	Years of follow-up	GRAF score	GRAF score association with fracture risk^1^
		Median (25%, 75%)	Median (25%, 75%)	HR (95% CI), p-value
ATI < 18 years cohort				
Total sample	656	7.9 (3.5, 13.9)	0.7 (0.4, 1.0)	
Patients w/ any type of fracture	33	4.5 (2.3, 8.6)	0.8 (0.7, 1.1)	All types of fracture 2.72 (1.14, 6.50), 0.0246
Patients w/spinal fracture	12	4.4 (1.3, 6.6)	0.9 (0.7, 1.2)	Spinal fracture 3.87 (0.92, 16.20), 0.0643
ATI ≥ 18 to < 50 years cohort				
Total sample	1485	13.8 (5.7, 20.1)	1.2 (0.9, 1.6)	
Patients w/ any type of fracture	167	6.4 (2.0, 13.7)	1.5 (1.2, 1.9)	All types of fracture 2.72 (2.06, 3.58), < 0.0001
Patients w/spinal fracture	61	6.0 (2.0, 12.9)	1.5 (1.3, 2.0)	Spinal fracture 3.44 (2.20, 5.38), < 0.0001
ATI ≥ 50 years cohort				
Total sample	507	8.2 (4.1, 14.3)	2.8 (2.3, 3.2)	
Patients w/any type of fracture	88	4.8 (1.7, 9.4)	3.1 (2.7, 3.6)	All types of fracture 2.72 (1.92, 3.84), < 0.0001
Patients w/spinal fracture	34	4.9 (1.1, 10.6)	3.1 (2.7, 3.6)	Spinal fracture 2.84 (1.62, 4.99), 0.0003

Abbreviations: ATI = age at treatment initiation; CI = confidence interval; GRAF = Gaucher Risk Assessment for Fracture; HR = hazard ratio

^1^HR and 95% CI estimated using Cox proportional hazards regression from age at treatment initiation (or age 18 years for patients initiating therapy prior to 18 years) to first fracture in adulthood, death, or end of follow-up. For each 1 unit increase in GRAF score, the fracture risk increases by 272% (HR = 2.72 across all ATI cohorts)

Each 1-point increase in GRAF score was associated with a 2.7-fold increased risk of first fracture (all types, *p* < 0.05) in adulthood for all 3 ATI cohorts (Table 2). An approximately 3- to 4-fold increased risk of spinal fracture was associated with each 1-point increase in GRAF score for all 3 ATI cohorts (Hazard Ratios = 2.84 to 3.87), which was significant (*p* < 0.001) for the 2 older ATI cohorts but not for the youngest ATI cohort (*p* = 0.06).

**Fig. 2:**
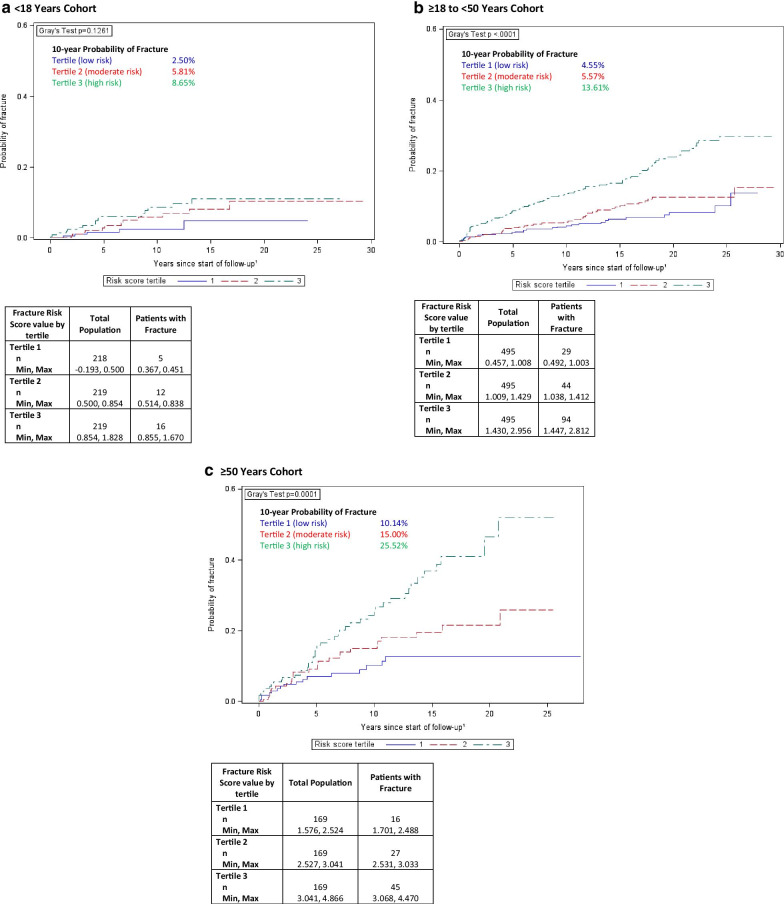
Cumulative incidence of total fracture by tertile of Gaucher Risk Assessment for Fracture (GRAF) score and by age at treatment initiation (ATI) cohort. For each tertile of the risk score, the cumulative incidence function (CIF) is plotted for all types of fracture. The CIF indicates the probability of a patient experiencing any type of fracture over time. The p-value from Gray’s Test indicates whether there is a significant difference (alpha level of < 0.05) between the 3 curves overall. Panel A includes the CIFs for the < 18 years ATI cohort; panel B includes the CIFs for the ≥ 18 to < 50 years ATI cohort; panel C include the CIFs for the ≥ 50 years ATI cohort. Follow-up starts at age 18 years for patients < 18 years at treatment initiation, and at age of treatment initiation for patients ≥ 18 years. The maximum value for the y-axis has been truncated from 1.0 to 0.6 as none of the CIFs went above 0.6

The cumulative incidence function plots (Fig. 2) illustrate the increasing probability of fracture over time based upon whether a patient’s GRAF score falls in the lower, middle, or upper third (i.e., tertiles) of the risk score values within each ATI cohort. Gray’s test, which assesses whether the cumulative incidence curves differed by GRAF score tertile, reached statistical significance for the 18 to < 50 ATI cohort (*p* < 0.0001) and the ≥ 50 year ATI cohort (*p* = 0.0001). It was not statistically significant, however, for the < 18 year ATI cohort (*p* = 0.13). For the < 18 year ATI cohort, the 10-year probability of fracture post-treatment initiation for the first, second, and third tertile was 2.5%, 5.8%, and 8.7%, respectively. For the ≥ 18 to < 50 years ATI cohort, the 10-year probability of fracture post-treatment initiation for each tertile was 4.6%, 5.6%, and 13.6%, respectively. For the ≥ 50 years ATI cohort, the 10-year probability of fracture post-treatment initiation for each tertile was 10.1%, 15.0%, and 25.5%, respectively.

The overall performance of the fracture risk model for predicting fractures as assessed by the integrated area under the curve (AUC) analyses was 0.62 for the < 18 years ATI cohort, 0.64 for the ≥ 18 to < 50 years ATI cohort, and 0.65 for the ≥ 50 years ATI cohort. Integrated AUCs were slightly higher for the spinal fracture risk model for all ATI cohorts (0.67, 0.68, and 0.67, respectively).

## Discussion

Traditional skeletal therapeutic goals for patients with GD1 concentrated on alleviation of bone pain, prevention of bone crises and osteonecrosis, and correction of osteopenia [19]. Many patients have now been treated for over 20 years and there is interest in formulating long-term therapeutic goals that emphasize positive functional outcomes. Such goals for the skeleton include maintenance of normal mobility and ability to exercise, and prevention of fractures and joint replacement [20]. Unfortunately, GD1 patients continue to suffer fractures as well as osteonecrosis despite years of otherwise effective ERT [21, 22]. The likelihood of post-ERT treatment bone complications is only partially predicted by either initial or serial GD disease severity scores (DS3) in which DXA score is a highly weighted component [22]. We investigated whether there are determinants for fracture other than DXA that can be easily identified when ERT is initiated and are of prognostic value for both treating physicians and patients.

We created a composite picture of fracture risk using the extensive data available in the large population of GD1 patients in the ICGG Gaucher Registry. The GRAF score uses variables easily accessible to the clinician and provides a tool to assess the relative risk of fracture within the GD1 population that may assist in management decisions. The current analyses combine important fracture risk factors relevant to the GD1 patient population and examine the impact of each factor, in the context of the other factors, through multivariable modeling and the calculation of a regression coefficient-weighted additive risk score. All types of first fractures occurring during adulthood and after initiation of imiglucerase treatment were evaluated for the association between the composite GRAF score and fracture risk. We limited our analysis of fracture risk to adults because the causes and anatomical distribution of fracture in children are different from those in adults. Patients who were never treated with ERT were excluded as they generally represent a distinct group with mild disease; however, the pre-treatment history of treated patients did inform the analysis. First spinal fractures during adulthood were also analyzed in a secondary analysis. It is important to note that comparisons of GRAF scores between ATI cohorts are not appropriate because the scores are based on the relative importance of the risk factors within each ATI cohort, and do not represent absolute measures such as incidence rate or cumulative incidence.

The well-recognized impact of age on fracture risk, particularly in post-menopausal women [23, 24] is accounted for by creating 3 cohorts based upon the age at which the patient first initiated imiglucerase treatment: < 18, ≥ 18 to < 50, and ≥ 50 years. In this population of GD1 patients, the average age of fracture (49.2 years) indicates that GD may accelerate the burden of fracture risk to occur sooner. Furthermore, the overall incidence rate of 9.4 fractures per 1000 person-years observed in our GD1 population is substantially greater than an age-adjusted incidence of approximately 1 per 1000 person-years for hip, vertebral and wrist fractures in a predominately white non-GD population from Minnesota USA [25]. The nearly 10-fold increase in fracture-incidence in our cohort of imiglucerase-treated patients also suggests that there are determinants specific to GD and the GD treatment journey that contribute to fracture risk. Our analysis identified 5 elements that may contribute to a high fracture risk in GD1 patients: sex, ATI, time between GD diagnosis and initiation of treatment, pre-treatment history of bone crisis, and pre-treatment splenectomy. After adjustment for all other factors in the multivariable model, the significance of each of these factors varied by ATI cohort. In the ATI < 18 year cohort, none of the individual factors were a significant indicator of fracture risk. In the middle cohort (ATI ≥ 18- < 50 y), all factors save sex were significant. In the ATI ≥ 50 year cohort, sex, age at treatment initiation, interval between diagnosis and treatment initiation, and history of bone crisis were significant factors; history of splenectomy was not significant in this subgroup.

Delayed initiation of treatment after diagnosis was consistently associated with increased risk for fracture in all patients who were older than 18 years when imiglucerase was begun. There was a 16 to 20% greater risk for fracture for every 10-year increase in duration between diagnosis and treatment initiation (hazard ratio: 1.16–1.20) (Fig. 1, panels B and C). In the patients in whom imiglucerase was begun before age 18 years, the interval between diagnosis and treatment is generally shorter than in the older patients [26]. There was no relationship between the time interval between diagnosis and initiation of treatment and fracture risk among patients treated before age 18. Ten years ago, data from the ICGG Gaucher Registry indicated that a delay in ERT initiation in excess of two years from diagnosis was associated with a significantly greater risk of developing osteonecrosis despite continuing treatment [27]. Our study is the first demonstration of delays in instituting ERT in GD1 patients whose disease severity ultimately warrants therapeutic intervention being associated with a sustained risk for future fractures. This risk increases with the interval between diagnosis and starting treatment, particularly for patients who initiate treatment in adulthood. In the older ATI cohorts, the longer time interval between diagnosis and treatment initiation could be indicative of GD patients with less severe hematological and visceral manifestations or they may have had a longer interval because they were diagnosed prior to the era when treatment was available. They then have a higher risk of fracture, possibly due to the progressive onset of “silent” bone disease manifestations in the absence of treatment.

GD1 patients with a history of total splenectomy prior to initiation of ERT are more prone to development of many late-onset complications including bone marrow failure, portal and pulmonary hypertension, osteolytic lesions, osteonecrosis and osteopenia [28]. The percentage of splenectomized patients in each of the 3 ATI cohorts is higher in patients who experienced fractures than in those without fractures (Table 1). Splenectomy was statistically significantly associated with fracture risk only in the ≥ 18 to < 50 years ATI cohort (56% greater risk of fracture post-splenectomy). The association between splenectomy and fracture risk in the < 18 years ATI cohort was a 61% greater risk of fracture post-splenectomy, but it did not reach statistical significance, possibly due to the small number of patients in this age group who had undergone splenectomy [26]. In the ≥ 50 years ATI cohort, the statistical analysis may have been affected by the exclusion of patients from the study population who may have experienced fractures during the long interval between the date of splenectomy and the date of initiation of imiglucerase.

Being female is a significant factor in the ≥ 50 years ATI cohort with presumably the highest proportion of post-menopausal women. Sex was not a significant factor for either of the two younger ATI cohorts. Our estimates of higher fracture risk in women compared to men over 50 years of age (approximately 2-fold) are in line with findings in the general population [29]. These findings suggest that general risk factors for post-menopausal osteoporosis (e.g. smoking, family history) also contribute to the overall fracture risk in female patients with GD1. Women of Ashkenazi Jewish ancestry have also been identified as being at high risk for fracture [30]. Although no subset analyses were conducted looking at ancestry due to the high proportion of patients without ancestry data reported in the Registry, women of Ashkenazi Jewish ancestry are a part of the population of this study and their risk is incorporated within the analyses for the GRAF score calculation. The refinement of the GRAF score with information about fracture risk in specific populations is a field for further study.

The GRAF score was significantly associated with fracture risk within each ATI cohort. In all cohorts, each 1-point increase in risk score was associated with a 2.7-fold increased risk of first fracture (all types) in adulthood. There was an approximately 3- to 4-fold increased risk of spinal fracture associated with a 1-point increase in risk score in all 3 ATI cohorts. The GRAF score was moderately successful in predicting fracture across the 3 ATI cohorts, as indicated by the integrated AUC (0.62, 0.64, 0.65). The results are comparable to AUC of the FRAX tool with post-menopausal women, both for FRAX and hip BMD (0.66 [95% CI 0.60–0.73]) and for FRAX alone (0.63 [95% CI 0.56–0.69]) [31, 32].

The cumulative incidence function plots illustrate the increasing probability of fracture over time based upon whether a patient’s risk score falls in the lower, middle, or upper third of the GRAF score distribution within each ATI cohort. The probability of fracture continually increases over the years of follow up. It does not plateau early after the start of ERT despite the fact that maximal hematological and visceral therapeutic responses are achieved within 5 years of initiation of treatment [33]. Significant differences in the cumulative incidence functions across the GRAF score tertiles were observed within the two older ATI cohorts, with the highest risk tertile appearing to have the greatest separation from the lower tertiles. The < 18 year ATI cohort did not show statistically significant differentiation among the tertiles; the moderate and high risk tertiles in this cohort appear to have the most similar trajectories. The scores that define each tertile within each ATI cohort are presented in Fig. 2. The instructions for calculating the risk score of individual patients are presented in supplemental materials (Additional file 1: S-I).

Although the GRAF score is derived from data of treated patients, the most appropriate time to apply the score may be at the decision point of initiating therapy. The age at treatment initiation and the interval between diagnosis and treatment initiation are important factors in the calculation. The physician faced with a treatment decision could apply the score based on the alternative assumptions that GD-specific treatment starts now (with current age and interval since diagnosis) or is deferred to a specific later date (when age and interval between diagnosis and start of treatment are clearly greater). The risk of post-treatment fracture under both scenarios may help the physician decide whether to recommend starting treatment or continue careful observation. However, the increased risk of fracture for those patients with a larger interval between diagnosis and treatment may also indicate that progressive skeletal disease has occurred during the untreated interval and delaying treatment further would not be indicated. A worked example is presented in supplementary materials (Additional file 1: S-I). The GRAF score addresses the association of deferment of treatment on the post-treatment risk for fractures. The score does not address the risk of fracture during any pre-treatment period, during which fracture risk is more appropriately estimated with serial DXA with or without FRAX scoring. Finally, there may be many other reasons besides fracture risk to treat GD; a low GRAF score alone does not necessarily indicate that treatment should be deferred.

The methodology behind the GRAF score is robust. The analyses show the impact of each factor while adjusting for all the other relevant factors, rather than estimating the association for each individual factor separately. Consequently, the GRAF score, which is calculated using the β estimates from the multivariable Cox proportional hazards regression (Additional file 1: S-I), is not a simple count of the number of risk factors. Instead it incorporates a weighting of each factor according to the strength of its association (i.e. the regression coefficient) with fracture risk. Patient age is an established risk factor for fracture [23, 24]. The GRAF score accounts for the increasing risk of fracture with age by stratifying patients within similar-aged peer groups based on age at treatment initiation and by including age at treatment initiation as a risk factor within each ATI cohort. The plots of the probability of fracture over time based on categories (i.e., lower, middle, and upper tertiles) of the GRAF score aid in the clinical interpretation of fracture probability for patients who fall into a certain GRAF score category. Using the instructions, equation, and β weights provided in supplemental materials (Additional file 1: S-I), a clinician can calculate a GRAF score for an adult, fracture-free patient based upon his or her age and select characteristics at treatment initiation. The probability of fracture over time can then be approximated using the cumulative incidence plots derived from the ICGG Gaucher Registry data.

Our study has limitations. The ICGG Gaucher Registry has enrolled a large international pool of over 6000 GD patients and has collected longitudinal data since 1991. However, it is a voluntary, observational database and missing data can be problematic. For this analysis, data sets are complete for all 3216 eligible patients. However, many patients were excluded because of incomplete diagnostic information or missing data about splenectomy status. We cannot be certain that these exclusions do not impact our results. We also cannot verify that all fracture events were reported to the Registry or that all fractures were due to fragility rather than excessive trauma. Data about concurrent illnesses, physical activity, history of falls, frailty, smoking, alcohol and drug use, family history of osteoporosis, and use of concurrent medications including anti-resorptive agents and vitamin D supplements might have been incorporated to improve the sensitivity and predictive ability of the GRAF score. However, this information is not collected in the ICGG Gaucher Registry. ERT dose was also not included in the score calculation due the complexity and potential incompleteness of the dose data. Attempting to incorporate all of these factors might also have rendered the GRAF score too complex for routine clinical use.

Because this study was restricted to patients treated with alglucerase and/or imiglucerase, we cannot assure that the GRAF score will necessarily be comparable for patients treated with other ERTs. In our study, 50 of all 288 first-time fractures (17%) occurred after a switch from imiglucerase to an alternative treatment. The impact of switching treatment on fracture risk is not known. No long-term head-to-head studies have been completed to support the hypothesis that there is no difference in therapeutic efficacy between imiglucerase, velaglucerase alfa and taliglucerase alfa. We are not aware of published data from the Gaucher Outcome Survey that primarily follows patients initially treated with velaglucerase regarding occurrence of fractures or osteonecrosis in its GD1 population. Pending such information, it seems likely to propose that GRAF scoring could reasonably be applied regardless of ERT choice [34]. Determining whether the GRAF score will accurately forecast fracture risk in treatment-naïve patients who are started on alternative, allegedly more bone-penetrant GD treatments (e.g., oral, small molecule substrate synthesis inhibitors) in adult GD1 patients will be of interest.

## Conclusions

The GRAF score is a tool to be used with bone density measurement and other modifiable, non-GD-specific risk factors (e.g., smoking, alcohol intake, frailty) to inform physicians and previously untreated GD1 patients about risk for a future fracture after starting imiglucerase or presumably, other alternative ERTS. GRAF scoring can also help predict the extent that fracture risk increases if initiation of treatment is deferred by either physician judgment or patient choice. Better prevention of serious bone complications is currently recognized as an unmet need for patients with GD1 [35]. When considering initiation of treatment, it is useful to know to what extent fixed patient-specific factors determine risk for future fractures beyond standard risk factors that change with time and treatment such as decreased bone mineral density. As similar risk factors determine the risk of post-treatment osteonecrosis [27], a risk-score based on the principles outlined in this paper could be applied to the likelihood of major bone events occurring: a combination of fracture and osteonecrosis, the two most impactful and feared complications of GD.

## Methods

### The ICGG Gaucher Registry

The ICGG Gaucher Registry is a voluntary, global, observational database with enrollment of over 6000 GD patients from more than 60 countries [36]. Patients’ treating physicians determine clinical assessments and care of enrolled patients, and the physicians voluntarily provide data on assessments of the clinical manifestations, GD progression and response to therapy.

This study was conducted according to the principles of the Declaration of Helsinki (2013) [37]. Informed consent was obtained from all subjects at the time of enrollment in the Registry. The data are reported in accordance with STROBE (Strengthening the Reporting of Observational Studies in Epidemiology) guidelines [38].

### Study population

Patients included in the study met all of the following criteria: (1) diagnosed with GD1 as determined by the participating Registry physician, (2) a known diagnosis date, a recorded birthdate, and a last follow-up date, (3) first primary therapy was imiglucerase, (4) had known splenectomy status and, if splenectomized, the date of the splenectomy was recorded, (5) at least one skeletal assessment recorded, and (6) if a fracture was reported, a date of fracture was recorded. All patients who met these inclusion criteria comprised the total study population for descriptive analyses.

For the risk factor analysis population, the sample was limited to patients with first fractures occurring after imiglucerase treatment initiation and in adulthood (age ≥ 18 years) as well as patients who did not experience a fracture during follow-up. Patients whose follow-up ended prior to 18 years of age or who experienced a fracture prior to treatment initiation or in childhood (age < 18 years) were excluded from the risk factor analysis.

### Definition of age at treatment initiation (ATI cohorts)

Patients were classified into 3 ATI cohorts based upon the age range of imiglucerase treatment initiation: 1) prior to 18 years of age, 2) between ≥ 18 and < 50 years, 3) ≥ 50 years of age. These age cohorts have been used in previous studies [26], including a GD1 bone study of the effect of alendronate sodium [39], and are designed to distinguish patients who began treatment early (during childhood or adolescence) as well as to distinguish women of post-menopausal age by using age ≥ 50 years as a proxy.

### Individual risk factors

Factors considered for inclusion in the analyses were based on the potential relationship with fracture and the availability and completeness of the data in the ICGG Gaucher Registry. Factors that met these criteria were sex, age at treatment initiation, time interval between diagnosis and treatment initiation, splenectomy status at treatment initiation, and history of bone crisis prior to treatment initiation. Sex was included due to the greater risk of fracture for post-menopausal women [18, 40]. The interval between diagnosis and treatment initiation has been associated with avascular necrosis [27] and may potentially be related to fracture risk. Splenectomy status was included because of lower bone density in those who have been splenectomized [41].

Other factors not in the current analysis include anemia (previous studies showed an association with avascular necrosis but not fracture [5]) and bone pain (there is no standardized scale between different centers, and bone pain and bone crisis are also highly correlated). Genotype was included initially but was not significant for any ATI cohort (data not shown) and was not included in the final model.

The importance of DXA BMD measurement for assessing hip and vertebral fracture risk specifically in patients with GD1 is well documented [5, 42, 43]. Because the GRAF score is static and subject to change only if treatment is not initiated at the time of calculation, it is intended to be used prior to DXA to assist the clinician with assessing to what extent the fracture risk will increase with additional delay and help the clinician with strategies to reduce risk of fracture. These strategies may include avoiding splenectomy, minimizing the time between diagnosis and treatment, considering the needs to optimize bone health in higher risk groups such as post-menopausal women, and treatment initiation prior to development of bone crises. Bone density from DXA rather than the GRAF score is more appropriate for assessing response and changing fracture risk after treatment initiation. For this reason, we decided not to attempt to incorporate the DXA or FRAX score into the GRAF.

### Derivation of the GRAF score

Cox proportional hazards regression was used to derive beta (β) coefficients for risk of first fracture (all types) separately by ATI cohort. Multivariable models included the 5 individual risk factors: sex (male, female), age at imiglucerase initiation (in years), time interval between diagnosis and imiglucerase initiation (in years), splenectomy status at treatment initiation (splenectomized, not splenectomized), and bone crisis ever reported prior to treatment initiation (yes, no). An additive summary risk score was calculated for each of the ATI cohorts by weighting the value of each risk factor by the corresponding β coefficient from the Cox model, where β represents the strength of the association for a particular risk factor. Additional details for calculating the risk score for an individual patient within a certain ATI cohort are described in supplemental material (Additional file 1: S-I).

### Statistical analyses

Descriptive analyses of demographic and clinical characteristics of patients with pediatric and adult first fractures and the distribution of fracture locations were conducted among the total study population. In addition, demographic and clinical characteristics of patients eligible for the risk factor analysis (i.e. patients who were fracture-free at baseline) were described. Baseline was defined as age 18 years for patients who initiated imiglucerase treatment < 18 years and as the date of imiglucerase initiation for patients who initiated treatment ≥ 18 years. The occurrence of multiple fractures on the first fracture date or subsequent fractures was also described among the risk factor analysis population.

For the analysis of individual risk factors for each ATI cohort, Cox proportional hazard regression was used to evaluate the multivariable model containing the 5 individual risk factors for risk of all types of fracture and then of spinal fractures separately. Patients were followed from baseline to the date of first fracture, death, or the last follow-up visit recorded, whichever occurred first. Patients may have switched or discontinued imiglucerase treatment during the follow-up period. Male was the referent category for sex. Splenectomy and bone crisis were scored as present or absent, with absence being the referent category. Age at treatment initiation and the time interval between diagnosis and treatment initiation were scaled to a 10-year interval for ease of interpretation; a 10-year difference is more clinically meaningful than a 1-year difference.

For the analysis of the GRAF score for each ATI cohort, Cox proportional hazard regression was used to evaluate the association between the continuous GRAF score and fracture risk (all types and spinal fracture). Patients were then classified into tertiles of risk score to plot the cumulative incidence of first fracture (all types) over time within each ATI cohort; Gray’s test was used to test whether the cumulative incidence function varied by risk score tertile. An integrated area under the ROC curve (AUC) was calculated to assess the ability of the risk score to classify patients by fracture status. The integrated AUC is a summary measure of the AUCs over the entire time period, which does not have the option of providing 95% confidence intervals (CI).

The primary hypothesis test for the association of GRAF score with fracture risk (all types) was two-tailed with an alpha level of 0.05, performed separately by ATI cohort. The association of GRAF score with spinal fracture risk was exploratory, so p-values were not corrected for multiple comparisons. Statistical analyses were conducted using the SAS software version 9.4 (Cary, North Carolina).

## Supplementary information

**Additional File 1.**  Gaucher Risk Assessment for Fracture Score Calculation Procedures.

**Additional File 2.** **Supplemental Table S-II**: Demographic and Clinical Characteristics for Patients with Fractures before Starting Treatment with Imiglucerase/Alglucerase, Pediatric and Adult Fractures

**Additional File 3.** **Supplemental Table S-III**: Demographic and Clinical Characteristics for Patients with Fractures after Starting Treatment with Imiglucerase/Alglucerase, Pediatric and Adult Fractures

**Additional File 4.** **Supplemental Table S-IV**: Skeletal Site Frequency for Pre-treatment Pediatric and Adult Fractures in GD1 Imiglucerase-treated Patients

**Additional File 5.** **Supplemental Table S-V**: Skeletal Site Frequencies of Pediatric and Adult First Fractures Occurring After Treatment Initiation among Imiglucerase-Treated GD1 Patients

**Additional File 6.** **Supplemental Table S-VI**: Descriptive Statistics for the Number of Fracture Sites Reported on the Dates of First and Subsequent Fractures Among Imiglucerase/Alglucerase-Treated ICGG Gaucher Registry Patients, Disease Type 1.

## Data Availability

The data that support the findings of this study are available from Sanofi Genzyme but restrictions apply to the availability of these data and are not publicly available. Data are, however, available upon reasonable request and with permission of Sanofi Genzyme.
