# Supplementary material for: The International Collaborative Gaucher Group GRAF (Gaucher Risk Assessment for Fracture) score: a composite risk score for assessing adult fracture risk in imiglucerase-treated Gaucher disease type 1 patients
Source: Orphanet J Rare Dis. 2021 Feb 18;16:92. doi: 10.1186/s13023-020-01656-6 (PMC7893749; doi:10.1186/s13023-020-01656-6)
Supplement: Supplementary file 1 — Additional File 1. Gaucher Risk Assessment for Fracture Score Calculation Procedures. [file 13023_2020_1656_MOESM1_ESM.docx]

**Gaucher Risk Assessment for Fracture (GRAF) score calculation**

**Below is a simplified graphic to assist in the calculation of the GRAF score. A more detailed explanation follows the graphic.**


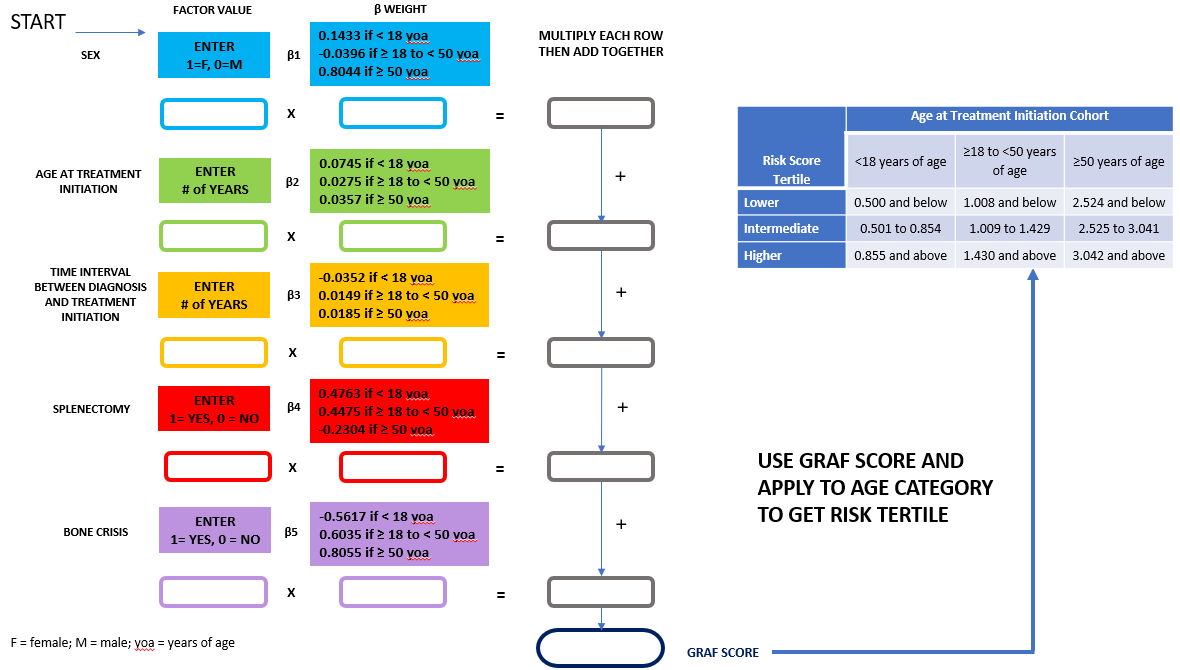


The GRAF score is an additive risk score calculated by weighting the presence or absence of each of 5 fracture risk factors (sex, age at treatment initiation, time interval between diagnosis and treatment initiation, splenectomy status, history of bone crisis) by the strength of their associations with fracture risk in adulthood, evaluated separately across 3 treatment initiation age groups: <18 years, ≥18 to <50 years, and ≥50 years of age.

**Deriving the beta estimates using the ICGG Gaucher Registry population:**

The study population used for the risk factor analysis is described in the Methods section of the manuscript. Cox proportional hazard regression was used to derive beta (β) coefficients for risk of first fracture (all types) separately by age at treatment initiation cohort. Multivariable models included the 5 individual risk factors: sex (female vs. male), age at imiglucerase initiation (in years), time interval between diagnosis and imiglucerase initiation (in years), splenectomy status at treatment initiation (splenectomized vs. not splenectomized), and bone crisis ever reported prior to treatment initiation (yes vs. no). The following table shows the β coefficients from the three multivariable models by each age at treatment initiation cohort in the ICGG Gaucher Registry population.

| **Risk Factor** | **Age at Treatment Initiation Cohort** | | |
| --- | --- | --- | --- |
|  | **<18 years of age** | **≥18 to <50 years of age** | **≥50 years of age** |
| Sex (ß1) | 0.1433 | -0.0396 | 0.8044 |
| AgeTxInitiation (ß2) | 0.0745 | 0.0275 | 0.0357 |
| TimeIntervalDxTx (ß3) | -0.0352 | 0.0149 | 0.0185 |
| Splenectomy ( ß4) | 0.4763 | 0.4475 | -0.2304 |
| BoneCrises (ß5) | -0.5617 | 0.6035 | 0.8055 |

Abbreviations: AgeTxInitiation = age at treatment initiation; TimeIntervalDxTx = Time interval between diagnosis and treatment initiation

**The GRAF score equation:**

**‘**GRAF SCORE’ for an individual patient is calculated according to his/her age category at treatment initiation cohort, using the beta estimates (ß1-ß5) from the corresponding Cox proportional hazards model shown in the table above, where ß represents the strength of the association for a particular risk factor with total fracture risk.

**GRAF SCORE** = (ß1*Sex) + (ß2*AgeTxInitiation) + (ß3*TimeIntervalDxTx) + (ß4*Splenectomy) + (ß5*BoneCrises)

**Steps to calculate the risk score for an individual patient:**

1. Determine the patient’s values for each of the 5 risk factors:
   1. For Sex, females are coded as 1 and males are coded as 0.
   2. For AgeTxInitiation, the value is the age in years at which the patient started treatment (imiglucerase/alglucerase).
   3. For TimeIntervalDxTx, the value is the number of years between the patient’s Gaucher disease 1 (GD1) diagnosis date and his/her treatment initiation date (imiglucerase/alglucerase). Subtract the age of diagnosis (in years) from the age of treatment initiation (in years).
   4. For Splenectomy, patients who were splenectomized prior to or on the date of starting imiglucerase/alglucerase treatment are coded as 1 and patients who were not splenectomized prior to or on the date of treatment initiation are coded as 0.
   5. For BoneCrises, patients with bone crises prior to or on treatment initiation date are coded as 1 and those without bone crises prior to treatment initiation are coded as 0.
2. Use the patient’s age at which s/he started imiglucerase/alglucerase therapy to determine which set of ß weights should be used to calculate the risk score.
3. Using the ß’s from the column in the table above that corresponds to the patient’s age at treatment initiation group, multiply each factor value by its associated ß, and then add the resulting values together to obtain the risk score.

**Determining a patient’s fracture risk score tertile based upon his or her calculated risk score and age at treatment initiation group:**

The table below shows the ranges of GRAF scores by tertile as calculated in the ICGG Gaucher Registry population.

| **Risk Score Tertile** | **Age at Treatment Initiation Cohort** | | |
| --- | --- | --- | --- |
|  | **<18 years of age** | **≥18 to <50 years of age** | **≥50 years of age** |
| **Lower** | 0.500 and below | 1.008 and below | 2.524 and below |
| **Intermediate** | 0.501 to 0.854 | 1.009 to 1.429 | 2.525 to 3.041 |
| **Higher** | 0.855 and above | 1.430 and above | 3.042 and above |

**EXAMPLES**

**Example calculation for a female patient who was diagnosed with GD1 at age 9, initiated imiglucerase treatment at age 11, did not have a splenectomy and did not experience a bone crisis event prior to initiation of treatment:**

**GRAF SCORE =** ß1*Sex + ß2*AgeTxInitiation + ß3*TimeIntervalDxTx + ß4*Splenectomy + ß5*BoneCrises = (0.1433*1) +(0.0745*11) + [(-0.0352)*2] + (0.4763*0) +[ (-0.5617)*0] = 0.8924

The patient would fall into the high fracture risk tertile group among patients who initiated treatment at <18 years of age.

**Example calculation for a female patient who was diagnosed with GD1 at age 23, initiated imiglucerase treatment at age 25, had a splenectomy, and experienced a bone crisis event at age 20:**

**GRAF SCORE** = ß1*Sex + ß2*AgeTxInitiation + ß3*TimeIntervalDxTx + ß4*Splenectomy + ß5*BoneCrises = [(-0.0396)*1] + (0.0275*25) + (0.0149*2) + (0.4475*1) + (0.6035*1) = 1.7287

This patient would fall into the high fracture risk tertile group among patients who initiated treatment between 18 and <50 years of age.

**Example calculation for a male patient who was diagnosed at age 50 and initiated imiglucerase treatment at age 50.5, did not have a splenectomy prior to treatment initiation and did not experience a bone crisis event prior to treatment initiation:**

**GRAF SCORE** = ß1*Sex + ß2*AgeTxInitiation + ß3*TimeIntervalDxTx + ß4*Splenectomy + ß5*BoneCrises = (0.8044*0) + (0.0357*50) + (0.0185*0.5) +[ (-0.2304)*0] + (0.5870*0) = 1.794

The patient would fall into the lower fracture risk tertile group among patients who initiated treatment ≥50 years of age.

**Example calculation using GRAF score to determine difference in fracture risk in 35-year-old treatment-naïve patient if treatment is initiated now or 10 years from now for a male patient diagnosed at 30 years, spleen intact, and no bone crisis.**

|  | **Initiate Treatment Now** | **Initiate Treatment 10 Years from Now** |
| --- | --- | --- |
| **GRAF Equation** | [(-0.0396)*0] + (0.0275*35) + (0.0149*5) + (0.4475*0) + (0.6035*0) | [(-0.0396)*0] + (0.0275*45) + (0.0149*15) + (0.4475*0) + (0.6035*0) |
| **GRAF score** | 1.037 | 1.461^*^ |
| **Risk score tertile** | Intermediate tertile | Highest tertile |

**^*^**The GRAF score increases to 2.065, also in the highest risk score tertile, if the patient experiences a bone crisis in the interim between this calculation and starting treatment. Bone crisis would possibly precipitate treatment.

Figure 2 in the manuscript plots the probability of fracture over time by risk score tertile and age at treatment initiation cohort. The second and third examples were designed, in part, to show how a similar GRAF score for two different patients will lead to a different estimation of risk for the patient depending upon the ATI cohort of the patient. It is also important to note that a particular patient’s individual fracture risk may differ from the ICGG Gaucher Registry patients’ risk as estimated in this analysis and may further vary according to other lifestyle and clinical factors not included in the GRAF score calculation. Because this study was restricted to patients treated with alglucerase and/or imiglucerase, we cannot assure that the GRAF score will necessarily be comparable for patients treated with other ERTs.
