# Supplementary material for: The International Collaborative Gaucher Group GRAF (Gaucher Risk Assessment for Fracture) score: a composite risk score for assessing adult fracture risk in imiglucerase-treated Gaucher disease type 1 patients
Source: Orphanet J Rare Dis. 2021 Feb 18;16:92. doi: 10.1186/s13023-020-01656-6 (PMC7893749; doi:10.1186/s13023-020-01656-6)
Supplement: Supplementary file 2 — Additional File 2. Supplemental Table S-II: Demographic and Clinical Characteristics for Patients with Fractures before Starting Treatment with Imiglucerase/Alglucerase, Pediatric and Adult Fractures [file 13023_2020_1656_MOESM2_ESM.docx]

Supplemental Table S-II: Demographic and Clinical Characteristics for Patients with First Fractures before Starting Treatment with Imiglucerase/Alglucerase, Pediatric and Adult Fractures

| Parameter | Statistics | Pre-treatment Pediatric Fractures | Pre-treatment Adult Fractures |
| --- | --- | --- | --- |
| Total Number of Patients with Fracture | N | 52 | 125 |
| Sex |  |  |  |
| Male | n (%) | 30 (57.7) | 53 (42.4) |
| Female | n (%) | 22 (42.3) | 72 (57.6) |
| Age at GD1 Diagnosis (years) |  | 52 | 125 |
|  | Mean (SD) | 14.2 (12.08) | 33.1 (19.09) |
|  | Median (25th, 75th) | 11.6 (5.0, 19.2) | 33.2 (19.0, 49.3) |
|  | Min, Max | 0.2, 58.5 | 2.0, 85.1 |
| Age at Imiglucerase Initiation (years) |  | 52 | 125 |
|  | Mean (SD) | 24.1 (14.23) | 47.6 (14.51) |
|  | Median (25th, 75th) | 19.5 (12.9, 31.7) | 47.4 (36.1, 57.2) |
|  | Min, Max | 1.2, 61.1 | 21.1, 87.1 |
| Age at First Fracture (years) |  | 52 | 125 |
|  | Mean (SD) | 10.2 (4.11) | 43.2 (15.75) |
|  | Median (25th, 75th) | 10.8 (7.3, 12.8) | 41.5 (30.5, 53.8) |
|  | Min, Max | 1.0, 16.8 | 19.4, 85.1 |
| Genotype |  | 52 | 125 |
| N370S/N370S | n (%) | 11 (21.2) | 31 (24.8) |
| N370S/Other | n (%) | 28 (53.8) | 68 (54.4) |
| Other/Other | n (%) | 9 (17.3) | 8 (6.4) |
| Unknown | n (%) | 4 (7.7) | 18 (14.4) |
| Splenectomized Prior to Date of First Fracture | n (%) | 10 (19.2) | 46 (36.8) |
