# Supplementary material for: The International Collaborative Gaucher Group GRAF (Gaucher Risk Assessment for Fracture) score: a composite risk score for assessing adult fracture risk in imiglucerase-treated Gaucher disease type 1 patients
Source: Orphanet J Rare Dis. 2021 Feb 18;16:92. doi: 10.1186/s13023-020-01656-6 (PMC7893749; doi:10.1186/s13023-020-01656-6)
Supplement: Supplementary file 3 — Additional File 3. Supplemental Table S-III: Demographic and Clinical Characteristics for Patients with Fractures after Starting Treatment with Imiglucerase/Alglucerase, Pediatric and Adult Fractures [file 13023_2020_1656_MOESM3_ESM.docx]

Supplemental Table S-III: Demographic and Clinical Characteristics for Patients with First Fractures after Starting Treatment with Imiglucerase/Alglucerase, Pediatric and Adult Fractures

| Parameter | Statistics | After Treatment Initiation Pediatric Fractures | After Treatment Initiation Adult Fractures |
| --- | --- | --- | --- |
| Total Number of Patients with Fracture | N | 52 | 288 |
| Sex |  |  |  |
| Male | n (%) | 25 (48.1) | 116 (40.3) |
| Female | n (%) | 27 (51.9) | 172 (59.7) |
| Age at GD1 Diagnosis (years) |  |  | 288 |
|  | Mean (SD) | 5.0 (3.16) | 24.7 (18.32) |
|  | Median (25th, 75th) | 4.2 (2.9, 7.2) | 21.4 (8.2, 37.0) |
|  | Min, Max | 0.2, 16.8 | 0.0, 78.9 |
| Age at Imiglucerase Initiation (years) |  |  | 288 |
|  | Mean (SD) | 7.2 (3.84) | 41.2 (17.08) |
|  | Median (25th, 75th) | 6.5 (4.3, 9.7) | 42.3 (29.6, 53.3) |
|  | Min, Max | 0.4, 17.5 | 3.1, 84.2 |
| Age at First Fracture (years) |  |  | 288 |
|  | Mean (SD) | 12.0 (3.98) | 49.2 (16.56) |
|  | Median (25th, 75th) | 12.6 (10.0, 15.0) | 49.5 (36.5, 62.0) |
|  | Min, Max | 0.9, 17.9 | 18.1, 90.4 |
| Genotype |  |  | 288 |
| N370S/N370S | n (%) | 2 (3.8) | 84 (29.2) |
| N370S/Other | n (%) | 29 (55.8) | 132 (45.8) |
| Other/Other | n (%) | 13 (25.0) | 42 (14.6) |
| Unknown | n (%) | 8 (15.4) | 30 (10.4) |
| Splenectomized Prior to Date of First Fracture | n (%) | 10 (19.2) | 121 (42.0) |

Note: 'Pediatric Fractures' refers to fractures occurring at age <18 years. 'Adult Fractures' refers to fractures occurring at age ≥18 years.
Note: The diagnosis date for one patient was set to the date of bone abnormality diagnosis.
