# Supplementary material for: The International Collaborative Gaucher Group GRAF (Gaucher Risk Assessment for Fracture) score: a composite risk score for assessing adult fracture risk in imiglucerase-treated Gaucher disease type 1 patients
Source: Orphanet J Rare Dis. 2021 Feb 18;16:92. doi: 10.1186/s13023-020-01656-6 (PMC7893749; doi:10.1186/s13023-020-01656-6)
Supplement: Supplementary file 4 — Additional File 4. Supplemental Table S-IV: Skeletal Site Frequency for Pre-treatment Pediatric and Adult Fractures in GD1 Imiglucerase-treated Patients [file 13023_2020_1656_MOESM4_ESM.docx]

Supplemental Table S-IV: Skeletal Site Frequency for Pre-treatment Pediatric and Adult First Fractures in GD1 Imiglucerase/Alglucerase-treated Patients

| **Parameter** | **Pre-treatment Pediatric Fractures**  **n (%)** | **Pre-treatment Adult Fractures**  **n (%)** |
| --- | --- | --- |
| Total Number of Patients with Fracture | 52 | 125 |
| Skeletal Site of First Fracture |  |  |
| Spine | 3 (5.8) | 33 (26.4) |
| Cervical Vertebra | 0 | 0 |
| Lumbar Vertebra | 0 | 2 (1.6) |
| Sacral Vertebra | 0 | 0 |
| Thoracic Vertebra | 0 | 0 |
| Coccygeal Vertebra | 0 | 0 |
| Vertebral Column, Unspecified | 3 (5.8) | 31 (24.8) |
| Femur/Hip | 7 (13.5) | 11 (8.8) |
| Femur, Distal | 1 (1.9) | 0 |
| Femur, Proximal | 1 (1.9) | 0 |
| Femur, Shaft | 0 | 0 |
| Femur, Unspecified | 4 (7.7) | 6 (4.8) |
| Hip Joint | 1 (1.9) | 5 (4.0) |
| Other | 6 (11.5) | 26 (20.8) |
| Pelvic Bone | 0 | 0 |
| Tibia | 0 | 4 (3.2) |
| Fibula | 0 | 1 (0.8) |
| Ankle Joint | 1 (1.9) | 0 |
| Foot | 0 | 2 (1.6) |
| Forearm | 0 | 2 (1.6) |
| Wrist Joint | 2 (3.8) | 1 (0.8) |
| Hand | 0 | 1 (0.8) |
| Rib | 0 | 7 (5.6) |
| Humerus, Proximal | 0 | 0 |
| Humerus, Distal | 0 | 0 |
| Humerus, Shaft | 0 | 0 |
| Humerus, Unspecified | 2 (3.8) | 4 (3.2) |
| Clavicle | 0 | 2 (1.6) |
| Knee Joint | 0 | 2 (1.6) |
| Elbow Joint | 0 | 0 |
| Shoulder Joint | 1 (1.9) | 0 |
| Jaw Joint | 0 | 0 |
| Skull | 0 | 0 |
| Unknown | 36 (69.2) | 55 (44.0) |

Note: “Pediatric Fractures” refers to fractures occurring at age <18 years. “Adult Fractures” refers to fractures occurring at age ≥18 years.

Note: One fracture site indicated per patient. If >1 first fracture site was reported, site was preferentially assigned according to the order listed in the table.
