# Supplementary material for: The International Collaborative Gaucher Group GRAF (Gaucher Risk Assessment for Fracture) score: a composite risk score for assessing adult fracture risk in imiglucerase-treated Gaucher disease type 1 patients
Source: Orphanet J Rare Dis. 2021 Feb 18;16:92. doi: 10.1186/s13023-020-01656-6 (PMC7893749; doi:10.1186/s13023-020-01656-6)
Supplement: Supplementary file 5 — Additional File 5. Supplemental Table S-V: Skeletal Site Frequencies of Pediatric and Adult First Fractures Occurring After Treatment Initiation among Imiglucerase-Treated GD1 Patients [file 13023_2020_1656_MOESM5_ESM.docx]

Supplemental Table S-V: Skeletal Site Frequencies of Pediatric and Adult First Fractures Occurring After Treatment Initiation among Imiglucerase/Alglucerase-Treated GD1 Patients

| **Parameter** | **After Treatment Initiation Pediatric Fractures**  **n (%)** | **After Treatment Initiation Adult Fractures**  **n (%)** |
| --- | --- | --- |
| Total Number of Patients with Fracture | 52 | 288 |
| Skeletal Site of First Fracture |  |  |
| Spine | 6 (11.5) | 104 (36.1) |
| Cervical Vertebra | 0 | 0 |
| Lumbar Vertebra | 0 | 11 (3.8) |
| Sacral Vertebra | 0 | 1 (0.3) |
| Thoracic Vertebra | 1 (1.9) | 18 (6.3) |
| Coccygeal Vertebra | 1 (1.9) | 0 |
| Vertebral Column, Unspecified | 4 (7.7) | 74 (25.7) |
| Femur/Hip | 9 (17.3) | 55 (19.1) |
| Femur, Distal | 0 | 4 (1.4) |
| Femur, Proximal | 2 (3.8) | 9 (3.1) |
| Femur, Shaft | 0 | 3 (1.0) |
| Femur, Unspecified | 4 (7.7) | 22 (7.6) |
| Hip Joint | 3 (5.8) | 17 (5.9) |
| Other | 28 (53.8) | 95 (33.0) |
| Pelvic Bone | 0 | 1 (0.3) |
| Tibia | 2 (3.8) | 11 (3.8) |
| Fibula | 0 | 3 (1.0) |
| Ankle Joint | 2 (3.8) | 8 (2.8) |
| Foot | 3 (5.8) | 18 (6.3) |
| Forearm | 7 (13.5) | 6 (2.1) |
| Wrist Joint | 3 (5.8) | 3 (1.0) |
| Hand | 3 (5.8) | 3 (1.0) |
| Rib | 2 (3.8) | 21 (7.3) |
| Humerus, Proximal | 0 | 1 (0.3) |
| Humerus, Distal | 0 | 0 |
| Humerus, Shaft | 0 | 2 (0.7) |
| Humerus, Unspecified | 1 (1.9) | 8 (2.8) |
| Clavicle | 3 (5.8) | 2 (0.7) |
| Knee Joint | 1 (1.9) | 5 (1.7) |
| Elbow Joint | 1 (1.9) | 3 (1.0) |
| Shoulder Joint | 0 | 0 |
| Jaw Joint | 0 | 0 |
| Skull | 0 | 0 |
| Unknown | 9 (17.3) | 34 (11.8) |

Note: “Pediatric Fractures” refers to fractures occurring at age <18 years. “Adult Fractures” refers to fractures occurring at age ≥18 years.

Note: One fracture site indicated per patient. If >1 first fracture site was reported, site was preferentially assigned according to the order listed in the table.
