# Supplementary material for: The International Collaborative Gaucher Group GRAF (Gaucher Risk Assessment for Fracture) score: a composite risk score for assessing adult fracture risk in imiglucerase-treated Gaucher disease type 1 patients
Source: Orphanet J Rare Dis. 2021 Feb 18;16:92. doi: 10.1186/s13023-020-01656-6 (PMC7893749; doi:10.1186/s13023-020-01656-6)
Supplement: Supplementary file 6 — Additional File 6. Supplemental Table S-VI: Descriptive Statistics for the Number of Fracture Sites Reported on the Dates of First and Subsequent Fractures Among Imiglucerase/Alglucerase-Treated ICGG Gaucher Registry Patients, Disease Type 1. [file 13023_2020_1656_MOESM6_ESM.docx]

| Supplemental Table VI: Descriptive Statistics for the Number of Fracture Sites Reported on the Dates of First and Subsequent Fractures Among Imiglucerase/Alglucerase-Treated ICGG Gaucher Registry Patients, Disease Type 1 | | |  |  |
| --- | --- | --- | --- | --- |
|  | | |  |  |
| Parameter | | Statistics | Patients with first fracture in adulthood | |
| N Patients | | N | 288 | |
| Number of fracture sites per patient reported on the first  fracture date | |  |  | |
| Continuous | | n | 288 | |
|  | | Mean (SD) | 1.1 (0.25) | |
|  | | Median (25th, 75th) | 1.0 (1.0, 1.0) | |
|  | | Min, Max | 1.0, 2.0 | |
| Categorical | |  |  | |
| 1 | | n (%) | 269 (93.4) | |
| 2 | | n (%) | 19 (6.6) | |
| 3+ | | n (%) | 0 | |
| Total number of all fracture sites per patient reported during  follow-up | | n | 288 | |
|  | | Mean (SD) | 1.7 (1.35) | |
|  | | Median (25th, 75th) | 1.0 (1.0, 2.0) | |
|  | | Min, Max | 1.0, 12.0 | |
| Total number of subsequent fracture sites per patient  reported after the first fracture date | |  |  | |
| Continuous | | n | 288 | |
|  | | Mean (SD) | 0.6 (1.35) | |
|  | | Median (25th, 75th) | 0.0 (0.0, 1.0) | |
|  | | Min, Max | 0.0, 11.0 | |
| Categorical | |  |  | |
| 0 | | n (%) | 199 (69.1) | |
| 1 | | n (%) | 53 (18.4) | |
| 2 | | n (%) | 18 (6.3) | |
| 3+ | | n (%) | 18 (6.3) | |
| Years from treatment initiation date to last follow-up date¹ | | n | 288 | |
|  | | Mean (SD) | 16.4 (7.27) | |
|  | | Median (25th, 75th) | 17.2 (11.1, 22.2) | |
|  | | Min, Max | 0.1, 28.1 | |

| ¹ For patients who initiated treatment at age <18 years, follow-up time was calculated from age 18 years onward. |
| --- |
